# Construction of a User-Led Resource for People Transitioning to Secondary Progressive Multiple Sclerosis: Results of an International Nominal Group Study

**DOI:** 10.3389/fneur.2020.00798

**Published:** 2020-08-18

**Authors:** Ambra Mara Giovannetti, Anna Barabasch, Andrea Giordano, Rui Quintas, Serena Barello, Guendalina Graffigna, Sara Alfieri, Insa Schiffmann, Cathleen Muche-Borowski, Claudia Borreani, Christoph Heesen, Alessandra Solari, the ManTra project

**Affiliations:** ^1^Unit of Neuroepidemiology, Fondazione IRCCS Istituto Neurologico Carlo Besta, Milan, Italy; ^2^Unit of Neuroimmunology and Neuromuscular Diseases, Fondazione IRCCS Istituto Neurologico Carlo Besta, Milan, Italy; ^3^Institute of Neuroimmunology and Multiple Sclerosis (INIMS), University Medical Center Hamburg-Eppendorf (UKE), Hamburg, Germany; ^4^Department of Psychology, University of Turin, Turin, Italy; ^5^EngageMinds Hub—Consumer, Food & Health Engagement Research Center, Department of Psychology, Università Cattolica del Sacro Cuore, Cremona, Italy; ^6^Unit of Clinical Psychology, Foundation IRCCS Istituto Nazionale per la Cura dei Tumori, Milan, Italy; ^7^Department of Neurology, University Medical Center Hamburg-Eppendorf (UKE), Hamburg, Germany; ^8^Department of General Practice/Primary Care, University Medical Center Hamburg-Eppendorf, Hamburg, Germany

**Keywords:** multiple sclerosis, conversion, secondary progressive multiple sclerosis, multiple-stakeholder consensus, nominal group technique

## Abstract

**Background:** ManTra is a mixed-methods, co-production research project for developing an intervention (resource) for people with newly diagnosed secondary progressive multiple sclerosis (pwSPMS) in Italy and Germany. In previous project actions, six resources were outlined, meeting the needs prioritized by pwSPMS.

**Aims:** This study aims to achieve multiple-stakeholder consensus on the most suitable resource and to refine the consensus resource.

**Methods:** Two nominal group technique (NGT) meetings were held, one in Milan and one in Hamburg. Participants were pwSPMS (five in Italy/six in Germany), pwSPMS significant others (SOs, four/five), healthcare professionals (HPs, seven/four), and health service researchers/patient and citizen organizations representatives (HPCORs, five/five). Two of the four resources discussed in each meeting were the same in Italy and Germany: “Promoting the engagement of pwSPMS: a program for the patients and the HPs” and “Enriched physiotherapy program for pwSPMS.” The other two were “A personalized care plan for pwSPMS” and “Roadmap for social and economic benefits” in Italy and “Metacognitive and everyday life training for pwSPMS” and “Psychological support for pwSPMS” in Germany. Each meeting consisted of two plenary sessions and a parallel group session (four stakeholder groups: pwSPMS, SOs, HPs, and HPCORs) in between. Meetings' narratives were analyzed thematically.

**Results:** The two meetings were rich in participation and discussion. In Italy, the consensus resource was “A personalized care plan for pwSPMS.” Refinements included enrichment with pwSPMS engagement, inclusion of additional HPs, improved definition of the MS nurse's role within the interdisciplinary panel, and community care integration. In Germany, the consensus resource was “Psychological support for pwSPMS.” Refinements included reshaping this resource into a more comprehensive and adaptive rehabilitation intervention and training the psychologist in recognizing client's rehabilitative needs and enhancing his/her autonomy.

**Conclusions:** The NGT eased multiple-stakeholder deliberation and resource fine-tuning in both countries.

## Introduction

About 15 years after diagnosis, around half of the people with relapsing–remitting multiple sclerosis (MS) develop secondary progressive MS (SPMS). This disease form is characterized by disability progression that is independent of a relapse, although people with SPMS (pwSPMS) can still experience relapses (1). Conversion from relapsing–remitting to SPMS is considered a key determinant of long-term disease prognosis. However, neither imaging criteria nor biomarkers are available to objectively distinguish relapsing–remitting from SPMS. SPMS is diagnosed retrospectively (2–4), and the period of diagnostic uncertainty may last for several years (5). A further challenge is presented by the scarcity of effective disease-modifying treatment options for pwSPMS (6, 7). Interferon β-1b and siponimod are the two treatments licensed by the EMA and only for people with active disease form.

Managing the Transition to SPMS (ManTra) is a mixed-methods project conducted in Italy and Germany that adheres to the Medical Research Council framework for developing and evaluating complex interventions (8). The project goals were 2-fold: to assess the experiences and the needs of people who recently converted to SPMS using qualitative and quantitative research and involving key stakeholders and to set up a user-led resource to empower and improve the quality of life and autonomy of newly diagnosed pwSPMS (9).

**Figure 1:**
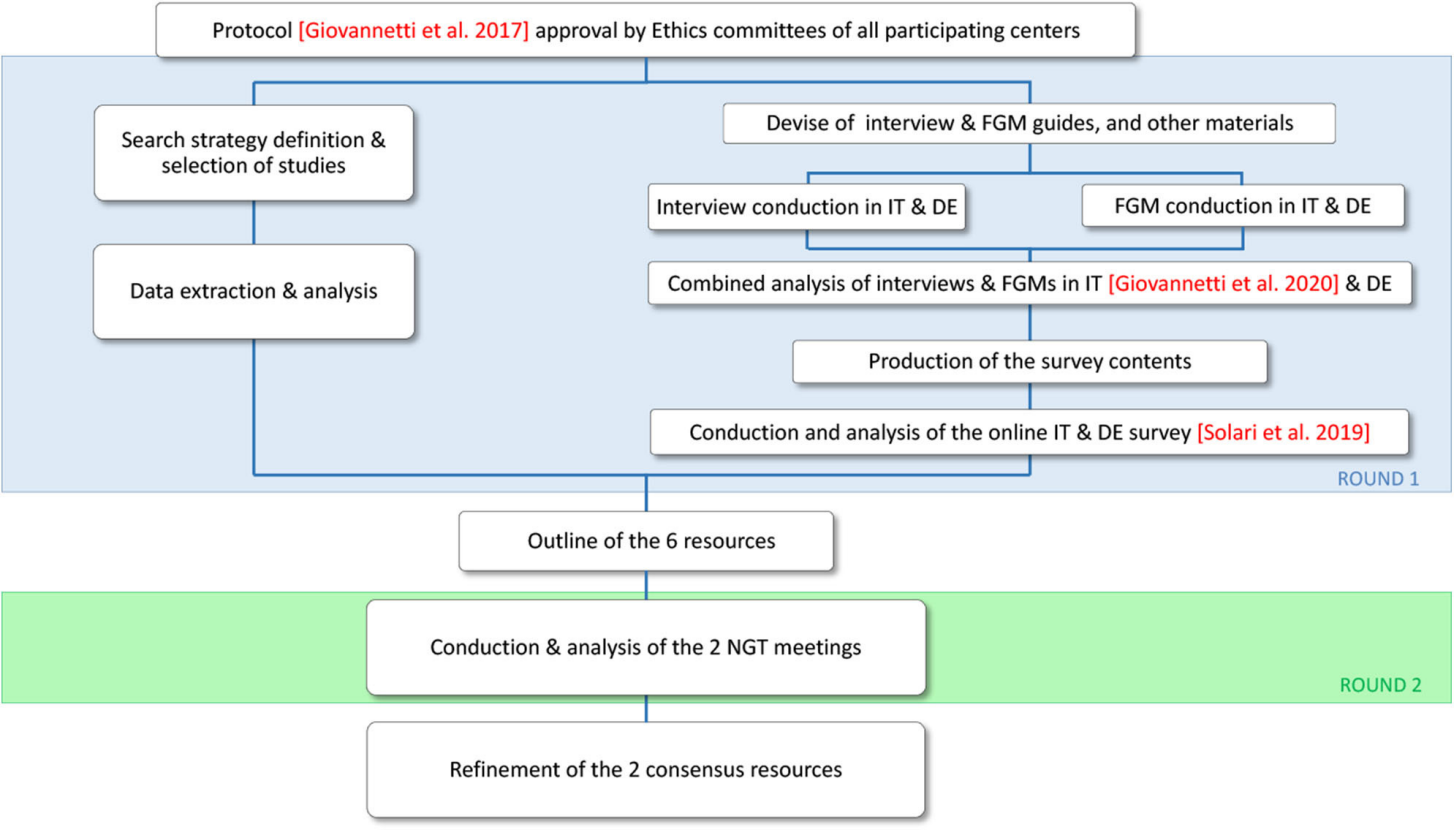
Flowchart of the ManTra Phase 1 project. The current study reports results of round 2 (green area). DE, Germany; FGM, focus group meeting; IT, Italy; NGT, nominal group technique.

In a previous round of the ManTra project (round 1; Figure 1), we identified 33 needs of people converting to SPMS via literature review and a qualitative study [personal semi-structured interviews with recently diagnosed pwSPMS; focus group meetings with pwSPMS' significant others (SOs), neurologists, and other healthcare professionals (HPs)] (10). An online survey with 215 recently diagnosed pwSPMS followed, assessing the characteristics associated with people's awareness of SPMS conversion, the experience of conversion, and the importance and prioritization of the 33 identified needs (11). Around 40% of survey participants were not aware of having SPMS (43% in Italy vs. 33% in Germany; *p* = 0.004). PwSPMS who were aware of their diagnosis were moderately to highly satisfied with the SPMS diagnosis disclosure. Activity limitations and geographic area were variables independently associated with pwSPMS awareness. Participants judged all the 33 needs identified as a lot to extremely important (11). The top four prioritized needs in Italy were “physiotherapy and exercise programs” (prioritized by 43% of survey participants), followed by “personalized care plan” (33%), “patient active involvement in care” (21%), and “information on social rights and policies” (17%). The top prioritized needs in Germany were “physiotherapy and exercise programs” (40%), “patient active involvement in care” (22%), “psychological support for patients” (22%), and “cognitive rehabilitation” (21%) (11).

For each prioritized need, the ManTra expert panel sketched out a dedicated resource. As two of the prioritized needs were shared by Italian and German pwSPMS, overall, there were six prioritized needs and six corresponding resources (Table 1). A detailed description of each consensus resource is provided in Supplementary File 1.

**Table 1:** The prioritized needs in Italy and Germany and the corresponding resources.

**Need**	**Country**	**Resource name, full**	**Resource name, short**	**Resource code**
An individualized healthcare plan	Italy	“A personalized care plan for pwSPMS”	“Personalized care”	Ai
Cognitive rehabilitation	Germany	“Metacognitive and everyday life training for pwSPMS”	Cognitive training”	Ag
Physiotherapy and exercise programs	Italy and Germany	“Enriched physiotherapy program for pwSPMS”	“Physio training”	B
Patient active involvement in healthcare	Italy and Germany	“Promoting the engagement of pwSPMS: a program for the patients and the HPs”	“Patient engagement”	C
Information on social rights and policies	Italy	“Roadmap for social and economic benefits”	“Rights and benefits”	Di
Psychological support for patients	Germany	“Psychological support for pwSPMS”	“Psychological support”	Dg

*pwSPMS, people with secondary progressive multiple sclerosis. The short resource name has been devised for the reader*.

The present paper reports the results of ManTra project round 2 (Figure 1), where a consensus on the most suitable resource to be assessed in each country was achieved using the nominal group technique (NGT) and resource refinements were performed.

## Materials and Methods

The ManTra project was approved by the ethics committees of the Fondazione IRCCS Istituto Neurologico Carlo Besta (clearance number: 27), the G D'Annunzio University of Chieti-Pescara (clearance number: 19), and the Aldo Moro University of Bari (clearance number: 98793CE) in Italy and the Hamburg Chamber of Physicians (clearance number: PV5733) in Germany. The resources outlined in round 1 were compared and prioritized in a 1-day meeting in each country using the NGT (12, 13).

### Participants

Each NGT meeting had 16–20 participants recruited from four stakeholder categories (four to five participants each): pwSPMS, SOs of pwSPMS, neurologists and other HPs caring for pwSPMS, and health service researchers and representatives of national patient and citizen organizations (HPCORs). Participants were selected using a purposive sampling technique. This sampling method is used in qualitative research where, as a rule, the number of participants is limited, and it is important to assure that the sample is as varied as possible in key features. To cover a range of experiences, they varied in terms of gender, education (pwSPMS and SOs), and neurological compromise (pwSPMS) (14). In Italy, participants were invited from different areas of the country.

PwSPMS were included if they were aged ≥18 years, diagnosed with SPMS from 3 months to 5 years (1), and fluent in Italian/German. PwSPMS with severe cognitive compromise (referring to neurologist's judgment) and those unable to communicate effectively were excluded.

SOs (relatives, partners, or close friends of a person who received a diagnosis of SPMS from 3 months to 5 years prior to inclusion) were included if they were ≥18 years old, provided emotional or tangible support to the pwSPMS during the SPMS disclosure period, and were fluent in Italian/German. Neurologists and other HPs were eligible if they were experienced in caring for pwSPMS and fluent in Italian/German.

### Procedure

The NGT is a highly structured method used for decision making within groups of experts (pwSPMS, families, people from the public, and stakeholders) (15). It requires direct participant involvement in a nonhierarchical way, and all participants have an equal voice and all responses to the posed question have equal validity (16).

In Italy, the NGT meeting was held at the Fondazione IRCCS Istituto Neurologico Carlo Besta, Milan. In Germany, it was held at the MS Day Hospital of the Institute of Neuroimmunology and Multiple Sclerosis, University Medical Center Hamburg-Eppendorf, Hamburg; each pwSPMS and SO received 25 euros for participation.

About 2 weeks before the meeting, participants were informed orally and in writing about the study purposes and procedures, and those agreeing to participate received an information pack detailing the four candidate resources (Supplementary File 1). Written informed consent was obtained from all participants before the meeting. Each meeting was structured in three sessions, guided by a moderator and four facilitators, and it was audio-recorded.

#### Plenary Session 1

The moderator explained the meeting's purpose and phases, explained the criteria to be used to prioritize the candidate resources, and emphasized the importance of each participant's contribution. She asked participants to introduce themselves and described each candidate resource neutrally, in order not to influence choices. Finally, each participant individually ranked each resource on a paper form for the following attributes: relevance, appropriateness (to people transitioning to SPMS), and ease of implementation (in clinical practice settings).

#### Parallel Group Session

Participants were divided into the four stakeholder groups (each working in a separate room) to facilitate going over the nominal group process in a group that feels on equal footing with each other. The facilitator first gave an overview of the individual ranking of Plenary Session 1, in order to detect common trends and divergences within the group members. Then each group discussed the resources guided by the facilitator (who took written notes) and ranked the candidate resources at a group level (overall appraisal). At the end of the session, the facilitator summarized the major discussion points and asked participants about any missed point.

#### Plenary Session 2

The facilitators presented the group ranking, and consensus on the most suitable resource was achieved via discussion. Further discussion of the consensus resource's contents and processes followed, guided by the moderator (who took written notes), in order to identify elements needing revision or refinement. Finally, the moderator summarized the major discussion points and asked participants about any missed point.

### Analysis

Descriptive statistics were calculated for general and clinical variables. Specifically, continuous variables were summarized by their mean and SD or median and range; categorical variables were summarized as numbers and percentages.

Narratives were analyzed thematically (17, 18). Our aim was to respect the cultural features and nuances of the source data by conducting the first (local) level of the analysis by a mother tongue researcher, followed by a second (combined) level, which was conducted in English. Specifically, AMG (Italy) and AB (Germany) collated and ordered the data according to themes, in order to enable comparison of comments from participants. Then, the two analyses were jointly discussed by AMG, AB, and AS. Meeting results were submitted to participants as a written report in Italy and as a PowerPoint presentation in Germany (respondent validation; Supplementary File 2). The reporting of the qualitative data follows the Consolidated Criteria for Reporting Qualitative Studies (Supplementary File 3).

## Results

The Italian team approached 25 experts by email and received 22 positive replies and three refusals (one pwSPMS and two SOs). One HPCOR participant from the South of Italy did not attend the meeting, which took place on November 30, 2018, between 10:30 a.m. to 4:30 p.m. The German team approached 55 experts (41 pwSPMS) by email or telephone and received 23 positive replies and eight refusals, while there was no reaction to 10 invitations. Three pwSPMS did not attend the meeting due to illness. The meeting took place on January 23, 2019 (3:00 to 6:00 p.m.). The characteristics of the participants are reported in Table 2.

**Table 2:** Characteristics of participants in the two nominal group meetings by country and stakeholder group.

	**Italy**				**Germany**			
**Characteristic**	**pwSPMS**	**SOs**	**HPs**	**HPCOR**	**pwSPMS**	**SOs**	**HPs**	**HPCOR**
	***N* = 5**	***N* = 4**	***N* = 7**	***N* = 5**	***N* = 6**	***N* = 5**	***N* = 4**	***N* = 5**
	***N (%)***							
Women	2 (40)	3 (75)	5 (71)	4 (80)	4 (67)	4 (80)	4 (100)	2 (40)
Age (years)^a^	50 (45–54)	38.5 (28–57)	46 (36–59)	45 (36–70)	58.5 (50–71)	47 (19–78)	51.0 (48–61)	53 (33–53)
**Area of Italy**								
North	4 (80)	3 (75)	5 (71)	1 (20)	–	–	–	–
Center	1 (20)	1 (25)	2 (29)	4 (80)	–	–	–	–
**Education**								
Degree/PhD	3 (60)	4 (100)	–	–	0	0	–	–
Secondary/high school	2 (40)	0	–	–	6 (100)	1 (20)	–	–
Primary school	0	0	–	–	0	4 (80)	–	–
**Occupation**								
Employed, fulltime	4 (80)	2 (40)	–	–	1 (17)	2 (40)	–	–
Employed, part-time	0	1 (20)	–	–	1 (17)	0	–	–
Student/housewife	0	1 (20)	–	–	0	1 (20)	–	–
Retired (disability)	1 (20)	0	–	–	3 (50)	0	–	–
Retired (age)	0	0	–	–	1 (17)	2 (40)	–	–
EDSS score^a^	6.0 (5.0–6.5)	–	–	–	4.25 (2.0–7.0)	–	–	–
Age at diagnosis of MS^a^	35 (23–39)	–	–	–	38 (32–40)	–	–	–
Years from SPMS diagnosis	2 (2–3)	–	–	–	4 (1–20)	–	–	–
**Relation with the patient**								
Husband/wife	–	3 (75)	–	–	–	2 (34)	–	–
Son/daughter	–	1 (25)	–	–	–	2 (34)	–	–
Mother	–	0	–	–	–	1 (17)	–	–
Expertise in MS (years)	–	–	20 (10–34)	12.5 (0–30)	–	–	18.5 (10–36)	20 (2–30)
MS pats followed (last 3 months)	–	–	60 (20–100)	–	–	–	40 (30–50)	–
SPMS pats follow (last 3 months)	–	–	20 (2–50)	–	–	–	15 (10–25)	–
**Profession**								
Neurologist/rehab physician	–	–	2 (28)	–	–	–	1 (25)	–
Nurse	–	–	1 (14)	–	–	–	1 (25)	–
Psychologist	–	–	2 (28)	–	–	–	1 (25)	–
Physiotherapist	–	–	1 (14)	–	–	–	1 (25)	–
Social worker	–	–	1 (14)	–	–	–	0	–

a *Median (range)*.

*EDSS, expanded disability status scale; HP, health professional; HPCOR, health service researchers and representatives of national patient and citizen organizations; pwSPMS, people with secondary progressive multiple sclerosis; SO, significant other*.

Both meetings were rich in participation and information, with discussions not only reflecting voting patterns but also adding understanding throughout. Sometimes, the multiple-stakeholder discussion was emotionally vivid and animated, but it was kept finalized by the moderator/facilitator, and the envisaged timeline was matched.

In both countries, participants had some difficulty in ranking the candidate resources using the three prespecified attributes: some had difficulty in discriminating between “relevance” and “appropriateness.” German pwSPMS had difficulty in ranking the resources for “ease of implementation” as they could hardly imagine how any of these interventions could be put into practice within the healthcare system. In addition, pwSPMS and SOs from both countries tended to remain anchored to their own needs (e.g., of physiotherapy and cognitive rehabilitation) in the ranking process, rather than considering the needs of other pwSPMS.

### Consensus Routing

#### Plenary Session 1

In Italy, ranking order (from highest to lowest) was “Personalized care”/“Physio training”/“Patient engagement”/“Rights and benefits” for relevance; “Physio training”/“Personalized care”/“Patient engagement”/“Rights and benefits” for appropriateness; and “Rights and benefits”/“Patient engagement”/“Physio training”/“Personalized care” for ease of implementation. In Germany, ranking order was “Physio training”/“Cognitive training” and “Psychological support”/“Personalized care” for relevance; “Psychological support”/“Cognitive training” and “Physio training”/“Patient engagement” for appropriateness; and “Psychological support”/“Cognitive training”/“Physio training”/“Patient engagement” for ease of implementation (Supplementary File 2).

#### Parallel Group Session

##### Italy

In Italy, both pwSPMS and SOs prioritized the resource “Physio training”; HPs prioritized “Personalized care”; and HPCORs did not achieve consensus between “Personalized care” and “Patient engagement,” which were both considered the most (and equally) important resources (Table 3). In Germany, pwSPMS, SOs, and HPCORs prioritized the resource “Psychological support”; pwSPMS agreed that both “Cognitive training” and “Psychological support” were the most (and equally) important resources; and HPs agreed that both “Physio training” and “Psychological support” were the most (and equally) important resources (Table 3).

**Table 3:** Priority rating of the parallel group sessions.

	**Italy**				**Germany**			
	**Ai**	**B**	**C**	**Di**	**Ag**	**B**	**C**	**Dg**
pwSPMS	2	1	3	3	1	2	3	1
SOs	2	1	2	3	3	2	4	1
HPCORs	1	2	1	3	3	2	3	1
HPs	1	2	3	4	2	1	3	1

*Ranking order: 1 (highest) to 4 (lowest). HP, health professional; HPCOR, health service researchers and representatives of national patient and citizen organizations; pwSPMS, people with secondary progressive multiple sclerosis; SO, significant other. Ai, “Personalized care plan for pwSPMS”; Ag, “Metacognitive and everyday life training for pwSPMS”; B, “Enriched physiotherapy program for pwSPMS”; C, “Promoting the engagement of pwSPMS: a program for the patients and the HPs”; Di, “Roadmap for social and economic benefits”; Dg, “Psychological support with the READY for MS program for pwSPMS”*.

Three stakeholder groups reached a consensus while HPCOR did not. The discussion flow is reported below. Reasons for choosing (or not) a resource as well as proposed resource improvements are reported in Tables 4, 5.

**Table 4:** Reasons for choosing a resource in Italy.

**Ai: “*Personalized care”***	**B: “*Physio training”***	**C: “*Patient engagement”***	**Di: “*Rights and benefits”***
• Includes resource B (pwSPMS)	• Important (pwSPMS)	• Empowers patients (pwSPMS and SO)	• Feasible (HPCOR)
• Interdisciplinarity as a plus (pwSPMS)/enhances interdisciplinary care (SO)	• Targets patient's functioning and autonomy (pwSPMS)	• Original (HPCOR)	• Concrete (HPCOR)
• Important (pwSPMS)	• Includes maintenance strategy (pwSPMS)	• Importance of involving HPs (HP training to offer patients a better service) (HPCOR)	• Easy to implement (SO)
• Innovative (HPCOR)	• Specific for people transitioning to SPMS (pwSPMS)	• Targets motivation (SO)	
• Tailored (using the diary) (HPCOR)	• Impacts both physical and mental health (SO)	• Impacts mental health (SO)	
• Specific for people transitioning to SPMS (SO and HPCOR)	• Is important for my relative (SO)	• Specific for people transitioning to SPMS (SO)	
• Promotes patient's empowerment (SO)	• Targets motivation (SO)	• Meets long-term needs (SO)	
	• Home rehabilitation as a value (SO)	• Suitable for each level of disease severity (SO)	

*HP, health professional; HPCOR, health service researchers and representatives of national patient and citizen organizations; pwSPMS, people with secondary progressive multiple sclerosis; SO, significant other*.

**Table 5:** Reasons for excluding a resource and (on the same line) possible improvements, in Italy.

**Ai: “*****Personalized care”***		**B: “*****Physio training”***		**C: “*****Patient engagement”***		**Di: “*****Rights and benefits”***	
**Reason**	**Improvem**.	**Reason**	**Improvem**.	**Reason**	**Improvem**.	**Reason**	**Improvem**.
• Hardiness of implementation (pwSPMS, HPCOR, and HP)	• Interactive tool may substitute face-to-face meeting (HPCOR)	• Already embedded in A (pwSPMS)	• Long-term rehab. program (pwSPMS)	• HP cultural resistance in participating (HPCOR)	• CME credits (HPCOR)	• The website is not interactive (HPCOR)	• Replace by an app (HPCOR)
• Time-consuming (HPCOR)	• Embed C in A (SO and HPCOR)	• Patient's risk of low adherence (SO)	• Personalized rehabilitation program (pwSPMS)	• Too much focus on HPs (HPCOR)	• Training should target patients, HPs, and institutions (HPCOR)	• Not relevant (pwSPMS and SOs)	
• It requires hospital–community connection (HPCOR)	• Creation of a dedicated MS clinic (HPCOR)	• It does not assure continuity (HP)		• “C” alone is not enough (HPCOR)	• The intervention should target an interdisciplinary team (HPCOR)	• Belongs to the MS society actions (no need for a dedicated research project) (pwSPMS, SO, HPCOR, and HP)	
• MS centers not able to take care of severely affected patients (HPCOR)	• Use the connection between hospital and community (HPCOR)			• Patient's involvement already exists (pwSPMS)	• It should be embedded in A (HPCOR)	• Easily embedded in the other resources (HP)	
• Diary may not suit well for all (HPCOR)	• Enrich the interdisciplinary team (HPCOR and HP)			• Not relevant (pwSPMS)			
• Motivation is not a target (SO)	• Use both diary and face-to-face meeting (HPCOR)			• Lack of psychological support (SO)			
• Similar to care pathway (HPs)	• Accessible website (HPCOR)			• Too much abstract (SO)			
	• HP training in communication (pwSPMS)			• Use of the web (SO)			

*CME, continuing medical education; HP, health professional; HPCOR, health service researchers and representatives of national patient and citizen organizations; pwSPMS, people with secondary progressive multiple sclerosis; SO, significant other*.

In the pwSPMS group, according to individual ranking (Plenary Session 1), resources “Physio program” and “Personalized care” obtained the highest scores, followed by “Patient engagement” and “Rights and benefits.” The discussion focused mainly on the two resources “Physio program” and “Personalized care.” pwSPMS stressed the importance of “Physio training,” and felt it is included in “Personalized care” as part of an individualized care plan. They reached a consensus on “Physio program,” mainly for the following reasons: it was judged crucial for pwSPMS functioning and autonomy and was more concrete and easier to implement than “Personalized care.”

“*If we want to obtain results that are immediate, practical, visible, and measurable, our choice is Resource B [‘Physio training’]. If we set up and run Resource B, in a short time we can show [to the scientific community] our findings, which should not be the case if we opt for Resource A [‘Personalized care’]. We can immediately obtain something concrete.”* [45 years old man, EDSS 6]

In the SO group, according to individual ranking, “Physio training” obtained the highest score, followed by “Personalized care,” “Patient engagement,” and “Rights and benefits” (equally preferred and consistently lower than “Physio training”). The resource “Rights and benefits” was considered as extremely easy to implement and of limited relevance, and it was quickly excluded. Resources “Personalized care” and “Patient engagement” were the most debated. Some believed “Patient engagement” was implicitly included in “Personalized care”; others perceived “Patient engagement” as superior to “Personalized care” because it focuses on pwSPMS engagement and autonomy. Because they could not find an agreement on one resource, they converged on “Physio training,” a resource that SOs knew was deemed important by their beloved. This stakeholder group did not provide information on ways to overcome the limitations identified in each resource.

“*I know for sure that B [‘Physio training’] is my husband first choice.”* [49 years old, woman]

In the HPCOR group, according to individual ranking (Plenary Session 1), “Personalized care” obtained the highest scores, followed by “Physio training” and “Rights and benefits” (equally preferred) and “Patient engagement.” The resource “Physio training” was quickly excluded from the discussion because it was perceived as part of the resource “Personalized care.” Resources “Personalized care” and “Patient engagement” were the most debated, while “Rights and benefits” faded in the background mainly because HPCORs felt it could be easily implemented by the patient's association without need for a dedicated research project. The HPCORs did not reach a consensus on a preferred resource between “Personalized care” and “Patient engagement.” They also agreed that “Patient engagement” should be included as a fundamental component of the resource “Personalized care.”

“*What I mean is … A [‘Personalized care’] is a great resource, for sure. Nevertheless, C [‘Patient engagement’] is essential to implement A. We will never put into action A without the information and education of all the stakeholders, that are the healthcare providers and the patients. So we must implement C first.”* [44 years old woman, researcher]

In the HP group, there were no resources that stood out at the beginning of the discussion. In fact, the resource “Personalized care” was on par with “Physio training” and “Patient engagement” in terms of relevance and appropriateness, while the resource “Rights and benefits” obtained high scores in ease of implementation. As in the HPCOR group, this resource quickly faded in the background. The HPCORs prioritized “Personalized care” and agreed that “Patient engagement” should be included in it as a fundamental resource's component.

“*It is unthinkable to run A [‘Personalized care’] without C [‘Patient engagement’]!*” [36 years old man, social worker]

##### Germany

Only the SO group reached a consensus on the prioritization of a single resource; all the other stakeholders rated as equally important at least two resources. Reasons for choosing or excluding the resource and possible improvements are reported in Tables 6, 7.

**Table 6:** Reasons for choosing a resource in Germany.

**Ag: “Cognitive training”**	**B: “*Physio training”***	**C: “*Patient engagement”***	**Dg: “Psychological support”**
• Extremely relevant (pwSPMS, SO, and HPCOR)	• Extremely relevant (pwSPMS, SO, and HPCOR)	• Importance of involving HPs (HP training to offer patients a better service) (SO and HPCOR)	• Relevant to strengthen resilience, acceptance, and mindfulness of the patient (pwSPMS, SO, HP, and HPCOR)
			• Relatives cannot take over the role of a psychologist (SO)
			• Basis for all other interventions (SO, HP, and HPCOR)

*HP, health professional; HPCOR, health service researchers and representatives of national patient and citizen organizations; pwSPMS, people with secondary progressive multiple sclerosis; SO, significant other*.

**Table 7:** Reasons for excluding a resource and (on the same line) possible improvements, in Germany.

**Ag: “Cognitive training”**		**B: “*****Physio training”***		**C: “*****Patient engagement”***		**Dg: “Psychological support”**	
**Reason**	**Improvem**.	**Reason**	**Improvem**.	**Reason**	**Improvem**.	**Reason**	**Improvem**.
• Patient transportation to the facility (SO)	• Replace face to face meetings with web tool (SO)	• Patient transportation to the facility (pwSPMS and SO)	• Finding a balance between excessive and insufficient training (SO)	• Focus on HPs and not on patients (HP)	• Training should target patients (HP)		Combination with:
• Difficult to integrate into healthcare according to preliminary studies (HP)	• Coverage of transportation costs by the healthcare insurance (SO)	• Patient's fatigue as a challenge (SO)	• If applicable, replace individual sessions with group sessions (HP)	• Too much effort for too little relevance (HP)	• Training of HPs in shared decision making rather than promoting pwSPMS engagement (HP)		• B (pwSPMS, SO, and HP)
	• Accessible website and no training (pwSPMS)	Difficult to implement (HP):	• Combination with B (SO and HP)	• HP resistance in participating and spending money for it (HP)	• Diary is not necessary (HP)		• Ag (pwSPMS)
	• Combination of A and D (pwSPMS)	• Training three times a week is too often			• Website with evidence-based information (HP)		• C (HPCOR)
		• pwSPMS have limited time as transition to SPMS around 45 years means possibly being employed and having children			• Training in patient autonomy in general and not only on SPMS (HP)		• Inclusion of Dg into a lifestyle intervention (HPCOR)
		• Limited evidence basis (HP)					• Accessible website
		• Access to physiotherapy is very good in Germany: B would be an extra offer (pwSPMS)					• Can be embedded in other resources (HPCOR)

*CME, continuing medical education; HP, health professional; HPCOR, health service researchers and representatives of national patient and citizen organizations; pwSPMS, people with secondary progressive multiple sclerosis; SO, significant other*.

In the pwSPMS group, according to individual ranking (Plenary Session 1), the resource “Cognitive training” received the highest scores, followed by “Psychological support,” “Physio training,” and “Patient engagement.” Nevertheless, in the parallel group discussion, all resources were perceived as important in the SPMS transition phase. At the end of the parallel group session, all pwSPMS equally preferred “Cognitive training” and “Psychological support,” followed by “Physio training” and “Patient engagement.”

“*A [‘Cognitive training’] and D [‘Psychological support’] on number 1! I could go with that.”* [55 years old woman, EDSS 6.0].

In the SO group, according to individual ranking (Plenary Session 1), the resource “Psychological support” received the highest scores, followed by “Cognitive training,” “Physio training,” and “Patient engagement.” After the parallel group discussion, the resource “Psychological support” was still prioritized, followed by “Physio training,” “Cognitive training,” and “Patient engagement.” “Psychological support” was seen as an important factor in the acceptance of the disease. Although “Physio training” and “Cognitive training” were regarded as very important resources, SOs felt that they both increase pwSPMS dependence on the social environment (it is usually up to SOs to bring pwSPMS to the facility where the training program is run).

“*In this case [‘Psychological support’], transport is less of a burden, because Dg [‘Psychological support’] is simply so relevant and the basis for everything else.”* [47 years old, wife]

In the HPCOR group, according to individual ranking (Plenary Session 1), “Psychological support” got the highest scores, followed by “Physio training.” “Cognitive training” and “Patient engagement” were rated equally in third place. This ranking was confirmed after the parallel group discussion. A key issue in the discussion was whether the priority between “Psychological support” and “Physio training” could be stated *a priori* or should depend on the specific context and on the pwSPMS situation in terms of both physical impairment and psychological skills. In line with this, it was emphasized that the engagement of the pwSPMS is a key ingredient for all resources.

“*It is not only important to provide the facilities [exercise courses, special counseling services etc.], but it is also very dependent on the patient-side which resource should have priority.”* [53 years old man, neurologist]

In the HP group, “Cognitive training” received the highest score in individual ranking (Plenary Session 1). “Physio training” and “Psychological support” were equally scored, followed by “Patient engagement.” After the group discussion, “Physio training” and “Psychological support” obtained equally the highest scores, followed by “Cognitive training” and then “Patient engagement.” HPs reported that providing psychological support to pwSPMS is more important than offering HP a training to promote patient engagement. Moreover, they expressed the concern that HPs in general might not be interested in participating in such a training, especially if they have to pay for it. One reason for not choosing “Cognitive training” was the difficulty encountered in implementing it at rehabilitation centers: At the inpatient level, a major issue is the health insurance company refund policy, which imposes a predefined rehabilitation plan; at the outpatient level, major issues are traveling to the rehabilitation facility and the time dedicated to the training sessions. Although “Physio training” was regarded as a very important resource, the whole design of the proposed program was questioned in terms of the lack of an evidence base supporting that individual sessions are more effective than group sessions and the scheduled number of sessions per week in the different phases:

“*B [Physio training] is not sufficiently founded in terms of expertise. The structure [i.e., the composition of an ‘Intensive phase’, ‘Extensive phase’ and a ‘Motivational component’] is not sufficiently justified by evidence. It should be piloted and possibly modified. The superiority of individual sessions is questionable and the frequency of the sessions seems too high.”* [61 years old woman, physiotherapist]

#### Plenary Session 2

In Italy, 19 of the 21 participants took part (one HP and one HPCOR left the meeting for work commitments). Two resources, “Personalized care” and “Physio training,” emerged as the most important (overall appraisal) in Italy. Participants agreed that the contents of the resource “Patient engagement” should be embedded into both “Personalized care” and “Physio training.” The choice between the two prioritized resources was difficult during the plenary discussion, which was vivid and passionate. At the end, the agreement was on “Personalized care.”

In Germany, 19 of 20 participants took part (one HP left the meeting for work commitments). All resources were regarded as very important (overall appraisal). All participants (18/19, one abstention) agreed on “Psychological support” after the plenary discussion.

##### Resource refinement

In Italy, participants suggested the following refinements to the resource “Personalized care”: (1) enrichment with the patient engagement component; (2) replacement of the term “multidisciplinary” with “interdisciplinary” and “multidimensional”; (3) inclusion of additional HPs (a physiotherapist and an obstetrician) in the interdisciplinary assessment; (4) improved definition of the role of the MS nurse in the process; (5) careful monitoring of each phase of the intervention to identify the most critical points. Participants identified three main resource challenges: (1) the use of the Canadian Occupation Performance Measure interview (http://www.thecopm.ca/) requires online certification, which can limit its wider use; (2) the processes described depend on the structural facilities available at a given center (e.g., dedicated slots for consultations and meetings); (3) they also depend on the extent of community care integration (hospital–community continuity).

In Germany, participants suggested to reshape the resource “Psychological support” into a more comprehensive and adaptive rehabilitation intervention. Specifically, the following refinements were proposed: (1) the psychologist should be specially trained to recognize the rehabilitative needs and priorities of each pwSPMS; (2) he/she should also be trained in enhancing pwSPMS empowerment and autonomy; (3) based on the initial comprehensive evaluation by the trained psychologist, timing of the psychological intervention should be carefully considered based on patient's need for metacognitive training and/or physiotherapy. The composition of the rehabilitation plan and the timing of each component should be tailored on patient's needs and priority.

### Respondent Validation

Feedback from participants on the meeting were positive in both countries. Worth mentioning is the critical appraisal of one Italian HPCOR (EUPATI patient expert). Two of her points were methodological: (a) The preference of pwSPMS was not fully valued, and patient choice should be weighted more than the choice of the other groups; and (b) the parallel group structure prevented an effective interaction between different stakeholders. In addition, she emphasized the implementation challenge of the consensus resource reported above (hospital–community continuity).

## Discussion

To our knowledge, this is the first study describing the development of a user-led resource for people transitioning to SPMS. Multiple stakeholders, including pwSPMS, their SOs, MS HPs, and HPCORs, were involved in resource prioritization and refinement. The NGT method was adopted to obtain consensus across stakeholders, who had equal opportunities of expressing their views and preferences. Research priorities were identified quantitatively (by ranking the candidate resources) and qualitatively (through critical reflection). As for the latter, discussion within and between stakeholder groups added understanding of voting patterns and provided hints for the refinement of the consensus resource in each country.

The ManTra project also exemplifies how such a research agenda might be implemented. The methods of the current and preceding project phases were coproduced and shared by the two countries, while the consensus meetings took place separately. This is because of the different languages (using English as *lingua franca* was not possible given the stakeholder groups involved) but also to respect the culture and the healthcare and social organization of the two involved countries.

In Italy, the selection of the resource “Personalized care” reveals the urgency of coordinated and interdisciplinary care, which becomes even more important as the disease worsens and impacts the personal and social life of the individual (10). Interestingly, in Italy, personalized care plan was the second prioritized need (33% of the online survey participants) in the ManTra online survey which preceded the present study (11), while in Germany, it was left out of the four prioritized needs, possibly reflecting differences in the organization of the healthcare system across the two countries (11). Nevertheless, it should be noted that most German participants were from the Hamburg metropolitan region, where barriers to integrated care pathways might be lower compared to those of small cities and rural areas.

In Germany, “Psychological support” was perceived as the priority in the transition phase. Participants believed that a psychological intervention may help pwSPMS cope with the high emotional burden due to the transition and support them to top up strategies to better deal with this new reality. Although Germany is among the countries with the highest number of psychotherapists, clinical experience supports the notion that psychological support is difficult to get for people suffering from chronic, physically disabling conditions and nearly never available on a home-based approach. In addition, mental symptoms are often seen as less worrying than physical symptoms by pwSPMS; they are perceived as an individual's matter, not a health problem, which leads pwSPMS to seek to conceal them (19). Finally, pwSPMS may hesitate to start a psychological support program or psychotherapy because of the fear to be stigmatized as being mentally ill (20). All these aspects demonstrated the urgency to develop a tailored intervention to answer the psychological needs of pwSPMS.

Exercise and physiotherapy programs were highly agreed-upon needs in Italy and Germany, even though they were not prioritized at the end of the NGT meeting. Although it has been recognized for over two decades that physiotherapy is beneficial for individuals with MS (21, 22), the MS barometer shows that access to rehabilitation varies widely across Europe, as theoretical approaches and quality standards (23). In Italy, physiotherapy continues to be a highly unmet need. In fact, it was the top prioritized need (43% of Italian participants) in the ManTra online survey which preceded the present study (11). In this country, access to outpatient physiotherapy in the public sector is limited to 40 sessions per year; multidisciplinary rehabilitation is at a premium, particularly through the public sector and in the South; and over one-quarter of Italian pwSPMS pay for rehabilitation out of pocket (24). In Germany, physiotherapy is available for a large number of patients, with some having one to two sessions of 20 min/week, but this format seems not to match pwSPMS needs. A recently published online survey of 212 MS physical therapists from 26 European countries shows that approaches differ largely between therapists, and except for northern regions, the general attitude is more “hands-on treatment” than “hands-off treatment” (providing advice and information) (25).

PwSPMS from the two countries showed some additional differences. While Italian pwSPMS were slightly younger and had shorter disease duration compared to German pwSPMS, disability was about the same in both groups. We do not think that differences in resource prioritization are based on these minor demographic and clinical differences but rather believe that they reflect sociocultural and healthcare organization differences.

A common issue across stakeholders and meetings was the suggestion to incorporate elements of the candidate resources into the prioritized one. This occurred particularly for the candidate resource “Patient engagement,” reflecting a need to respect and enhance pwSPMS autonomy, independently from the resource type. There was a considerable consensus in Italy and Germany about the need of programs to promote an active role among pwSPMS. However, this finding might also reflect the influence of the research team, as the ManTra project investigators have been conducting research in MS shared decision making for over a decade.

## Limitations

As mentioned above, the ManTra project was led by two teams of researchers working in the field of shared decision making. In addition, most of the German pwSPMS were from the Hamburg area, and they were active in self-help organizations and in other patient associations. In addition, only pwSPMS with the mental capability to participate in the NGT meeting were included. Thus, they might not be representative of pwSPMS at large.

One German pwSPMS was far from the transition phase as he had SPMS for 20 years (protocol violation), although in a rather stable form.

Despite these limitations, it is important to notice that we applied purposive sampling to achieve a broad range of general and clinical characteristics (EDSS scores between 2.0 and 7.0). Moreover, due to the structured and nonhierarchical approach, all participants/stakeholders were given a voice in the meeting, pointing to issues that may have been previously unidentified (16, 26).

## Conclusions

The present study allowed us to select and refine a user-led resource to deal with SPMS transition in Italy and Germany. The NGT we used permitted us to take into consideration each individual and stakeholder point of view. The discussion allowed us to arrive to a consensus on a single resource and provided key input for resource improvement. In Italy, the priority was a personalized healthcare plan for pwSPMS, while in Germany psychological support was deemed as the most important resource to implement. In addition, the study offers a step-by-step guidance on how the guided consensus can be managed at an international level, by involving participants with different cultures and languages. The methodology used can be easily exported in other contexts/countries and, we believe, applied to different health conditions.

## Data Availability Statement

The raw data supporting the conclusions of this article will be made available by the authors, without undue reservation.

## Ethics Statement

The studies involving human participants were reviewed and approved by Fondazione IRCCS Istituto Neurologico Carlo Besta ethics committee. The patients/participants provided their written informed consent to participate in this study.

## Author Contributions

AS, AGiov, AGior, and CB conceived the NGT study of the ManTra project. AS, AGiov, and AGior wrote the NGT study protocol. AS, AGiov, AGior, RQ, and SA ran the NGT meeting in Italy. CM-B and AB ran the NGT meeting in Germany. AGiov, AB, and AS conducted the qualitative analysis. AS, AGiov, AB, and CH drafted the manuscript. All authors contributed to the article and approved the submitted version.

## Collaborators

Steering Committee: Claudia Borreani, Giovanna De Luca, Andrea Giordano, Ambra Mara Giovannetti (study Co-PI), Lara Gitto, Christoph Heesen, Alessandra Solari (study PI), Valentina Torri Clerici, Maria Trojano, Michele Messmer Uccelli.

Literature Review Panel: AG, AMG, Andrea Fittipaldo, Sascha Köpke.

Qualitative Analysis Panel: Anna Barabasch, CB, AMG, Erika Pietrolongo.

Expert Panel: AB, AG, AMG, LG, EP, MMU, IS, CT, VTC. The panel, appointed to outline and revise the candidate resources, consisted of a researcher (health economist) who has had MS for 15 years (LG), a psychologist/methodologist (AG), three neurologists (CT, VT, and IS), three MS clinical psychologists (AMG, AB, and EP), and a nurse/AISM delegate (MMU).

Online Survey Panel: AB, AG, AMG, IS, MMU.

Centers and investigators: Fondazione IRCCS Istituto Neurologico Carlo Besta, Milan: Unit of Neuroepidemiology, Giusi Ferrari, AF, Arianna Fornari, AG, AMG, AS; Unit of Neuroimmunology and Neuromuscular Diseases: Paolo Confalonieri, AMG, VTC; Department of Neuroscience, Imaging and Clinical Sciences, G D'Annunzio University of Chieti-Pescara, Chieti: GDL, Deborah Farina, Marco Onofrj, EP; Departments of Basic Medical Sciences, Neurosciences and Sense Organs, Aldo Moro University of Bari, Bari: Pietro Jaffaldano, Alessia Manni, MT; Unit of Psychology, Foundation IRCCS Istituto Nazionale per la Cura dei Tumori, Milan: Sara Alfieri, CB; Italian Multiple Sclerosis Society and Research Foundation (AISM), Department of Health Services and Research, Genova: MMU; CEIS Economic Evaluation and HTA, Università degli Studi di Roma “Tor Vergata,” Rome, Rome: LG. Department of Neurosciences, San Camillo Forlanini Hospital, Rome, Italy: Maria Esmeralda Quartuccio, CT. Institute of Neuroimmunology and Multiple Sclerosis, Department of Neurology, University Medical Center Hamburg-Eppendorf, Hamburg, Germany: AB, CH, Anne Christin Rahn, IS. Kempfenhausen Center for Treatment of Multiple Sclerosis, Marianne-Strauß-Klinik, Berg, Germany: Ingo Kleiter.

## Conflict of Interest

AS reports grants from Fondazione Italiana Sclerosi Multipla (FISM), during the conduct of the study; personal fees from Biogen Idec, Merck Serono, Novartis, Almirall, and EXCEMED. RQ received funding for conducting research and clinical projects from AIM Education (Novartis) and Fondazione Serono (Merck) in 2019. AB has received funding from Roche Pharma. CH has received research grants, congress travel compensations, and salaries for talks from Biogen, Genzyme, Sanofi-Aventis, Bayer Healthcare, Merck, Teva Pharmaceuticals, Roche Pharma, and Novartis. The remaining authors declare that the research was conducted in the absence of any commercial or financial relationships that could be construed as a potential conflict of interest.
