# Supplementary material for: Construction of a User-Led Resource for People Transitioning to Secondary Progressive Multiple Sclerosis: Results of an International Nominal Group Study
Source: Front Neurol. 2020 Aug 18;11:798. doi: 10.3389/fneur.2020.00798 (PMC7461961; doi:10.3389/fneur.2020.00798)
Supplement: Supplementary file 1 [file Data_Sheet_1.PDF]

Supplementary File 1. Description of the candidate resources (English version).

frontiers

Title of the resource:

A personalized care plan for people with SPMS (Resource Ai)

In response to the need:

A personalized care plan…

Description of the resource (intervention contents)::

The plan consists of 4 phases.

- **Phase 1: Baseline interview**

The person with secondary progressive multiple sclerosis (SPMS) and, if he/she desires, a family member, are invited to an initial interview that aims to take stock of the situation and start planning a multidisciplinary evaluation. The neurologist and possibly another member of the MS Center team participate in the interview.

- **Phase 2: Multidisciplinary functional assessment and goal setting**

Evaluation: Six professionals will be involved (consultations). Some of them will also systematically consider the need to involve other professionals, shown in brackets:
1. neurologist (ophthalmologist)
2. physiatrist (occupational therapist, speech therapist, nutritionist, technical evaluation aids)
3. urologist (sexologist)
4. neuropsychologist
5. psychologist (psychiatrist, sexologist)
6. social worker

Each professional will produce a written consultation report containing the summary of the evaluation carried out and the results of the tests performed. This report will be loaded on a dedicated platform (ManTra electronic platform).

Goal setting: a specially trained professional of the MS Center will conduct a structured interview with the patient to identify the objectives relevant to him/her, allowing the caring team to take these objectives into consideration at each stage of the process. To do this, the operator will use a specific tool, the *Canadian Occupational Performance Measure (COPM)*, which assesses the functioning of the person's daily, social and work activities.
This phase will be completed within 3 months.

- **Phase 3: Multi-disciplinary discussion and draft of the personalized care plan**

The professionals involved in the multi-disciplinary functional assessment meet (in person or via teleconference) to discuss the patient situation and identify the best possible interventions. Specifically, they focus on: assessment of the clinical problems, the needs and priorities of the patient (identified with the COPM), proposed program with detailed priority setting, resources already in place and those to be activated. As a result, a written *personalized care plan* is drafted.

- **Phase 4: Restitution and refinement of the personalized care plan**

The patient (alone or with a family member) and the caring neurologist (together with another MS team member if indicated) share and discuss the *personalized care plan* draft at the MS Center through: a) an interview; b) a written text detailing and time-lining the consultations /exams / therapies to be performed. Based on the interview, the plan is perfected and the final version ('action planning') is produced.

ManTra electronic platform

An electronic platform will be created containing the patient's documentation, the *personalized care plan*, the visits and treatments envisaged in it. To each player (professionals, patient and family), a password will be provided for access to the platform. Through the platform, the patient (and if desired, also the family member) will receive notifications on date / time / place of the visits / therapies / rehab sessions to be carried out consistently with the plan.

Title of the resource:

Metacognitive and everyday life training for pwSPMS (Resource Ag)

In response to the need:

Access to cognitive rehabilitation

Description of the resource (intervention content):

Fatigue, depression and problems with thinking and memory (so-called cognitive problems) are common symptoms of multiple sclerosis (MS) and are of great importance for social participation and occupation of patients. The following programme aims to treat cognitive and psychological impairments.

Step 1: Cognitive and psychological testing

- Evaluation of daily neuropsychological functions
- Questioning/participation of relatives

Step 2: Feedback and treatment development, discussion of regular MS rehabilitation, neuropsychological therapy in practice

Step 3: Metacognition and training

3.a Metacognitive and Everyday Life Training for MS Patients (MaTiMS)

The MaTiMS program was developed for the treatment of cognitive and psychological (neuropsychiatric) impairments. The focus is on the patients' own perception of these impairments. It aims to treat the most important neuropsychiatric impairments in MS: memory, attention, depression, fatigue, stress and social empathy.

Sessions are conducted in small groups of 5-8 participants. Trainers (psychologists or trained MS nurses) teach the following topics in six independent modules (duration: approx. 90 minutes, once a week): attention, memory, fatigue, depression, stress and social empathy.

MaTiMS includes an a) psycho-educative and a b) interactive part with examples and exercises.

a) Information based on the current state of scientific knowledge, on typical symptoms, epidemiological data and factors leading to those symptoms, as well as on therapeutic options. A moderator accompanies and guides the group through each module and gives examples. Participants have the opportunity to report their experiences and explain their own coping strategies.
b) The exercise part revives the examples. Possible mistakes or wrong approaches in dealing with deficits are explained and suggestions for better coping strategies, thoughts and concepts are presented. The participants' experiences are also central to this: they can explain their own behaviour in dealing with deficits and point out successful experiences. Especially the direct exchange of strategies, experiences, opinions and thoughts between the participants helps to recognise, rethink and change one's own non-functioning behaviour patterns. Here the participants also receive homework, which they can do with the help of a brochure with

training contents, which should help to reflect the learned elements and to transfer them into everyday life.

Relatives support the training by being included into the homework

3b: Computer-based working memory training

In a cognitive computer training (e.g. BrainStim) the participant works independently with a predefined plan and a training brochure after an introduction by a psychologist. The program has the ability to adapt the level of difficulty of the training to the individual performance of the patient. The training focuses on attention, linguistic and spatial memory. The training is carried out twice a week for 6 weeks at 45 minutes each.

Step 4: Computer- and web-based support program

An electronic platform will be created containing documentation for and by the patient, the personalised care plan, the visits and treatments. Each participant (professional, patient and relative) will be provided with a password to access the platform. Via the platform, the patient (and, if desired, the relative) receives notifications of the date / time / place of visits / therapies / rehabilitation sessions, which are carried out in accordance with the plan.

Expected benefits:

- Perception of one's own cognitive performance through testing
- Better self-management and development of coping strategies through metacognition and computer training
- Long-term support through computer platform
- Greater satisfaction with one's own performance and thus more self-confidence in coping with requirements and deficits

Title of the resource:

Enriched physiotherapy program for pwSPMS (Resource B)

In response to the need:

Access to physiotherapy and exercise programs …

Description of the resource (intervention contents):

The program consists of an intensive outpatient phase, followed by an extensive home-based phase, 'enriched' by a motivational intervention.

• **Intensive outpatient phase**

The rehabilitation program will focus on improving walking and reducing fatigue.
A "circuit training" is provided, which includes three treatment stations:
1) "*Treadmill training*" with walking and balance exercises in which the participant is asked to maintain a certain level of physical effort calibrated according to his/her clinical needs.
2) *Balance exercises* on the ground, in standing position and in transfer (eg passage from sitting upright), varying the size of the support base, the sensory context (e.g. closed eyes), the movements of the head and eyes.
3) *Muscle strengthening exercises* for the impaired muscles, in particular the extensor muscles of the lower limbs, the tibialis anterior and the middle buttocks. The exercises will be performed through the use of elastic bands or weights anchored to the body segments.
This phase will last for 8 weeks / 24 sessions (3 sessions a week of 45-60 minutes). The activities will be performed on an outpatient basis with the physiotherapist in a 1: 1 ratio (individual sessions).

• **Extensive home-based phase**

The rehabilitation program will focus on the same functions as the previous phase. Walking exercises, static equilibrium and reinforcement are planned. A manual will be used for the patient self-executed exercises and for the detection/monitoring of the activities carried out. The rehab team will select the type and intensity of the exercises according to the clinical characteristics of the individual patient.
This phase will last of 16 weeks / 32 sessions (2 sessions a week of 30-45 minutes each), and will be divided into 4 phases (a-d), each lasting 4 weeks:
a) *Program setting and self-performed exercises ('self')* - First and second week: 8 individual sessions with the therapist. Third and fourth week: 4 sessions with the therapist alternating with 4 self-executed sessions.
b) *Self-Phase 1* - The patient will self-execute the exercise program established in the previous phase (8 sessions). Adherence will be assessed by telephone follow-up at the completion of the first and third week.
c) *Program re-setting and self* - In 4 weeks the patient will perform 4 sessions with the therapist alternating with 4 self-executed sessions.
d) *Self-Phase 2* - The patient will self-execute the exercise program established in the previous phase (8 sessions). Adherence will be assessed by telephone follow-up at the completion of the second and fourth weeks.

• **Motivational intervention**

The objective of this component is twofold: a) the promotion of the active participation of the patient in the definition of the goals of the treatment ('goal setting') and their achievement; b) a transfer of skills, with behavioral change to an increased motor activity in everyday life. The motivational intervention will start from the intensive phase and throughout the extensive phase. The *Canadian Occupational Performance Measure (COPM; http://www.thecopm.ca/)* will be used to define the objectives of the treatment. The reference theory is the 'COM-B Framework' developed by Susan Michie. According to this model, voluntary behavior is the result of the individual's capacity (physical and psychological), external opportunities (physical and social), and motivation (reflexive and automatic). The type and schedule of the motivational intervention are being defined.

Expected benefits:

- → Although the efficacy of neuro-rehabilitation in people with MS has been consolidated, the follow-up of the studies is short and it is not known when to re-schedule a new intensive phase. In this study, which includes a one-year follow-up and quarterly visits, it will be possible to produce scientific evidence regarding this important aspect.
- → The study will make it possible to determine the role of the extensive phase in lengthening the benefits gained from the intensive phase (primary research objective).
- → It will also allow to evaluate the additional effect of the motivational component.

Title of the resource:

Promoting the engagement of people with SPMS: a program for the patients and the HPs (Resource C)

In response to the need:

Active involvement of the patient/SO in the care pathway

Resource description (intervention contents)

People with chronic illnesses who are not actively involved in the treatment process are at higher risk of disease worsening than those with a high level of involvement. It is therefore key to promote: 1) greater knowledge of patient own illness; 2) active involvement of the patient in everything concerning his/her care path (patient engagement).
The ultimate goal is to foster autonomy and proactivity of the person in the management of his/her lifestyle and health care.

To achieve this goal, the present resource consists in two actions, one directed at the health providers (HPs) and one directed at the people with SPMS and their significant others:

• Action 1: HP training to patient engagement
The training course is aimed at MS Center HPs, each group consisting of 10-15 participants. Training strategies include lectures, exercises and experiential practices. There are two phases: 1) initial training and 2) maintenance phase.

Phase 1 - Initial training
The training includes the following contents:
• Values, skills and principles of patient engagement (2h)
• The Shared Decision-Making model (2h)
• Exercises for the development of skills and competences on the points above (7h)
• Reflections on care, assistance and promotion of well-being in people with SPMS

Phase 2 - Maintenance (duration 6 months)
In this phase the implementation of patient engagement skills will be supported in HP work context through:
• a monthly e-mail with some practical advice
• A monthly telephone contact with an experienced patient engagement operator to support the use of strategies and skills in clinical activity

• Action 2: A website dedicated to the person with SPMS
Parallel to the conduct of the training it will be set up dedicated website where the person with SPMS (and his/her family) can access to improve their skills on specific topics related to the treatment and management of the disease.

The contents of the website will be determined by:

• Identification of the main topics (eg change/termination of immunotherapy, symptoms, lifestyle) by conducting focus group meetings and personal semi-structured interviews with people with SPMS and their family members.
• Systematic search of the scientific evidence related to each topic.

The structure of the website will consist of:
• Chapters, each structured in an introduction on the topic, the intervention / treatment options and the benefits and risks associated with each option.
• A diary in which the patient reports the type and severity of his/her symptoms and problems. The diary will have a structured section (including validated questionnaires), and an open section for comments and notes. It can be printed out to share contents during consultations at the MS center.

Expected benefits:
→ Improvement of the skills of the patient (and his/her family) in relation to the SPMS phase and possible treatments
→ Improvement of HP skills in the active involvement of the patient (and his/her family) in the treatment process and in decisions
→ Decision-making that combines the best available scientific evidence and the values and preferences of the individual patient
→ Improvement of the quality of care, treatment adherence, and of continuity of care

frontiers

Title of the resource:

Roadmap for social and economic benefits (Resource Di)

In response to the need:

Information on social rights and policies

Description of the resource (contents of the intervention):

Rights and benefits are a wide-ranging and complex topic. Available information tools are too general in contents, or puzzling. For people with MS and their families it is important to clarify what are the most important needs for the specific situation they are facing at a given moment, and be guided to the most appropriate solution without getting lost in the complexity of bureaucracy and legislation. This requires a dynamic tool, able to adapt to the different needs and expectations.

The proposed resource consists of two on-line components: an information tool and a section for dialogue and sharing ('Community').

1. **The information tool** is structured as a roadmap that aids the identification of the most correct solution, according to a guided orientation flow. The theme of the roadmap concerns the benefits associated with the disability and of working facilities (in Italy, Law 104). The user (the patient or relative) is asked to provide information on his/her situation, with the aim of being guided, step by step, to the contents most relevant and specific to his/her situation. Based on the replies made by the user to items on his/her disease and living condition, the orientation flow helps to define the specific need related to the stage of life or disease course. Once the need has been clarified, the system guides the user to the most coherent information resources.

2. **The Community** allows the peer exchange of information on relevant topics and practical issues, within the Community of people with MS and their family members. A Community moderator gathers specific requests and directs the user towards competent professional operators.

Through the informative tool and the Community, users can be directed for obtain further information from professional operators of the Italian MS Society (AISM) toll-free number (social worker and labor consultant lawyer), or to the reception and information desks of the AISM local sections.

Expected benefits:

- → People with MS and their families will have access to personalized information and support on the subject of social rights and policies
- → Opportunity to share experiences and obtain information from peer exchange

The online format eases timely updating of the information

Title of the resource:

Psychological support for pwSPMS (Resource Dg)

In response to the need:

Access to psychological support for patients

Description of the resource (intervention content):

Step 1: Psychological testing

Psychological assessment with questionnaires and interviews (2-3 sessions, cooperation with psychological counselling centre of the German Multiple Sclerosis Association

Step 2: Feedback, counselling and treatment planning, offers (3.a)

- Identify the challenges the patient faces in terms of disease progression, family/role adaptation and working life
- Evaluation of the patient's competences by the treatment team
- Decision on type and scope of disease management

Step 3.a: Acceptance and commitment therapy (ACT) in MS (READY for MS)

ACT is a newer form of psychotherapy that combines classical behavioural techniques with mindfulness and acceptance-based strategies.

The READY for MS programme aims to improve resilience, mental flexibility, physical activity, mindfulness, subjective well-being, health behaviour and quality of life. READY is based on the core processes of ACT: acceptance, mindfulness, cognitive defusion (changing attitudes to unpleasant thoughts), mindfulness, the observing self, values and committed action.

READY for MS consists of a) 7 weekly modules of 2.5 hours each and a b) booster session. It is offered as a group program for 5 to max. 12 participants.

a) Weekly modules with psychoeducation, experience exercises and structured learning and exercise activities.

1) Introduction to the READY resilience model

2) Mindfulness (feeling the “here and now”)

3) Defusion (dealing with unwanted thoughts)

4) the observing self (solutions to assumptions about oneself, change of perspective)

5) Acceptance (willingness to accept all sensations)

6) Values (what is really important) and meaningful actions, social networking, pleasant activities (active action)

7) Committed action (dealing with potential problems)

Participants receive a detailed information book with written notes, sections for critical reflection, structured learning activities and a CD with guided mindfulness meditation exercises. With selected reflection and learning exercises, an individual READY plan is created to help participants apply the generalized information in their specific context and individual environment.

b) A booster session 5 weeks after the last session should refresh the contents of the individual modules and address implementation problems.

Step 3.b:

- Personal psychotherapy (if necessary), cooperation with the psychotherapist Chamber, development of a MS contact list

or

- Computer-assisted/telephone therapy (MS-DEPREXIS)

Step 4: Computer- and web-based support program

An electronic platform will be created containing documentation for and by the patient, the personalised care plan, the visits and treatments. Each participant (professional, patient and relative) will be provided with a password to access the platform. Via the platform, the patient (and, if desired, the relative) receives notifications of the date / time / place of visits / therapies / rehabilitation sessions, which are carried out in accordance with the plan.

Emergency telephone/email consultation by a therapist

Expected benefits:

- Increasing the psychological flexibility required for a value-oriented life, which constantly faces changing internal and external living conditions.

- Improvements in the areas of resilience, mindfulness, subjective well-being, health behaviour and quality of life.

- Developing coping strategies
