# Supplementary material for: Construction of a User-Led Resource for People Transitioning to Secondary Progressive Multiple Sclerosis: Results of an International Nominal Group Study
Source: Front Neurol. 2020 Aug 18;11:798. doi: 10.3389/fneur.2020.00798 (PMC7461961; doi:10.3389/fneur.2020.00798)
Supplement: Supplementary file 2 [file Data_Sheet_2.PDF]

Supplementary File 2. NGT reports for respondent validation

Report Progetto ManTra – Meeting di consenso

30 Novembre 2018

Sede dell'incontro: AmadeoLab

Inizio:10.45

Termine: 16.30

Partecipanti: 5 persone con sclerosi multipla, 4 familiari, 7 operatori, 5 ricercatori/rappresentanti delle associazioni di pazienti.

Moderatrici: Ambra Mara Giovannetti, Alessandra Solari

Facilitatori: Sara Alfieri, Andrea Giordano, Ambra Mara Giovannetti, Rui Quintas

1. Prima Plenaria

Nella prima fase dell'incontro, AS ha presentato le quattro risorse identificate come prioritarie durante l'indagine online condotta su scala nazionale e coordinata dal Servizio di Neuroepidemiologia della Fondazione IRCCS Istituto Neurologico C. Besta. I contenuti delle risorse sono stati trasmessi ai partecipanti una settimana prima dell'incontro, sotto forma di schede sinottiche allegate a questo documento.

Di seguito sono elencati i titoli delle quattro risorse presentate:
(Ai) Piano di cura personalizzato per le persone con sclerosi multipla secondariamente progressiva;
(B) Programma integrato di riabilitazione motoria per le persone con sclerosi multipla secondariamente progressiva;
(C) Promuovere il coinvolgimento attivo delle persone con sclerosi multipla secondariamente progressiva: un programma diretto agli operatori sanitari e ai pazienti;
(Di) Roadmap per le agevolazioni sociali e economiche.

Al termine di questa fase, a ciascun partecipante è stato chiesto di prioritizzare ciascuna risorsa in base ai seguenti criteri:

Rilevanza: quanto ritieni importante questa risorsa.
Appropriatezza: quanto ritieni che la risorsa proposta sia adeguata a rispondere al bisogno descritto.
Facilità di attuazione: quanto ritiene che la risorsa sia attuabile nella pratica clinica.

Risultati della Prima Plenaria:
Di seguito viene riportato l'ordine di prioritizzazione delle risorse rispetto a ciascun criterio:
Rilevanza (dalla più rilevante alla meno rilevante): Ai B C Di
Appropriatezza (dalla più appropriata alla meno appropriata): B Ai C Di
Facilità di attuazione (dalla più attuabile alla meno attuabile): Di C B Ai

2. **Gruppi in parallelo**

I partecipanti sono stati divisi in gruppi omogenei (1. Persone con sclerosi multipla secondariamente progressiva, SMSP; 2. Familiari di persone con SMSP; 3. Operatori sanitari; 4. Ricercatori/rappresentanti delle associazioni). In ciascun gruppo uno psicologo ha facilitato le attività finalizzate al raggiungimento di un consenso rispetto alla prioritizzazione delle risorse.
In ciascun gruppo il facilitatore ha fornito al gruppo una sintesi dell'andamento delle valutazioni, mostrando eventuali punti di convergenza e divergenza tra i membri del proprio gruppo. Il gruppo ha poi avviato una fase di confronto con l'obiettivo di raggiungere un consenso interno.

Risultati Gruppi in parallelo
Persone con sclerosi multipla e loro familiari hanno identificato come prioritaria la risorsa B *(Programma integrato di riabilitazione motoria per le persone con sclerosi multipla secondariamente progressiva)*, gli operatori sanitari la risorsa A, mentre i ricercatori/rappresentanti delle associazioni non hanno raggiunto un consenso, giudicando come prioritarie sia la risorsa A che la C.

3. **Seconda Plenaria**

Durante questa fase due partecipanti (un operatore sanitario e un rappresentante delle associazioni) hanno dovuto lasciare la riunione per precedenti impegni lavorativi. I partecipanti sono quindi scesi da 21 a 19.
In base ai risultati della fase precedente, le risorse A e B sono risultate le più rilevanti e appropriate; è stato suggerito di inserire i contenuti della risorsa C in A e B; la risorsa D è stata descritta come la più facile da attuare, ma anche la meno rilevante e appropriata. La D è stata quindi esclusa dalla votazione finale. La risorsa A è stata scelta come prioritaria.
Ai partecipanti è stato poi chiesto di fornire suggerimenti per migliorare questa risorsa. Di seguito vengono riportati i contenuti emersi:

a. Arricchire la risorsa, inserendo una componente sull'engagement del paziente;
b. Aggiungere ulteriori figure professionali nella valutazione interdisciplinare (in particolare fisioterapista, ostetrico);
c. Definire in modo più chiaro e preciso il ruolo dell'infermiere;
d. Porre attenzione all'integrazione ospedale-territorio;
e. Valutare l'utilizzo o meno del *Canadian Occupation Performance Measure* (misura di esito) per

la definizione degli obiettivi condivisi. Il fatto che richieda una certificazione online potrebbe limitarne la diffusione;

f. Utilizzare il termine ‘interdisciplinare’ e ‘multidimensionale’ al posto di multidisciplinare;
g. Monitorare le tempistiche di effettuazione di ogni fase dell’intervento;
h. Prevedere le modifiche strutturali/organizzative necessarie affinché i processi descritti siano realmente attuabili (ad es. prevedere e verificare la disponibilità di slot dedicati per le consultazioni e le riunioni).

In conclusione, i partecipanti hanno raggiunto il consenso rispetto alla prioritizzazione della risorsa A, (Piano di cura personalizzato per le persone con sclerosi multipla secondariamente progressiva), fornendo anche suggerimenti per il suo miglioramento.

German NGT report for respondent validation

UKE HAMBURG

INIMS Institute of Neuroimmunology and Multiple Sclerosis

Diskussionsrunde

Managing the transition (ManTra): Eine Ressource für Menschen mit sekundär progredienter Multipler Sklerose

23.01.2019

Fondazione I.R.C.C.S. Istituto Neurologico Carlo Besta

Sistema Socio Sanitario Regione Lombardia

SCLEROSI MULTIPLA fondazione italiana ONLUS

un mondo libero dalla SM

Teilnehmende

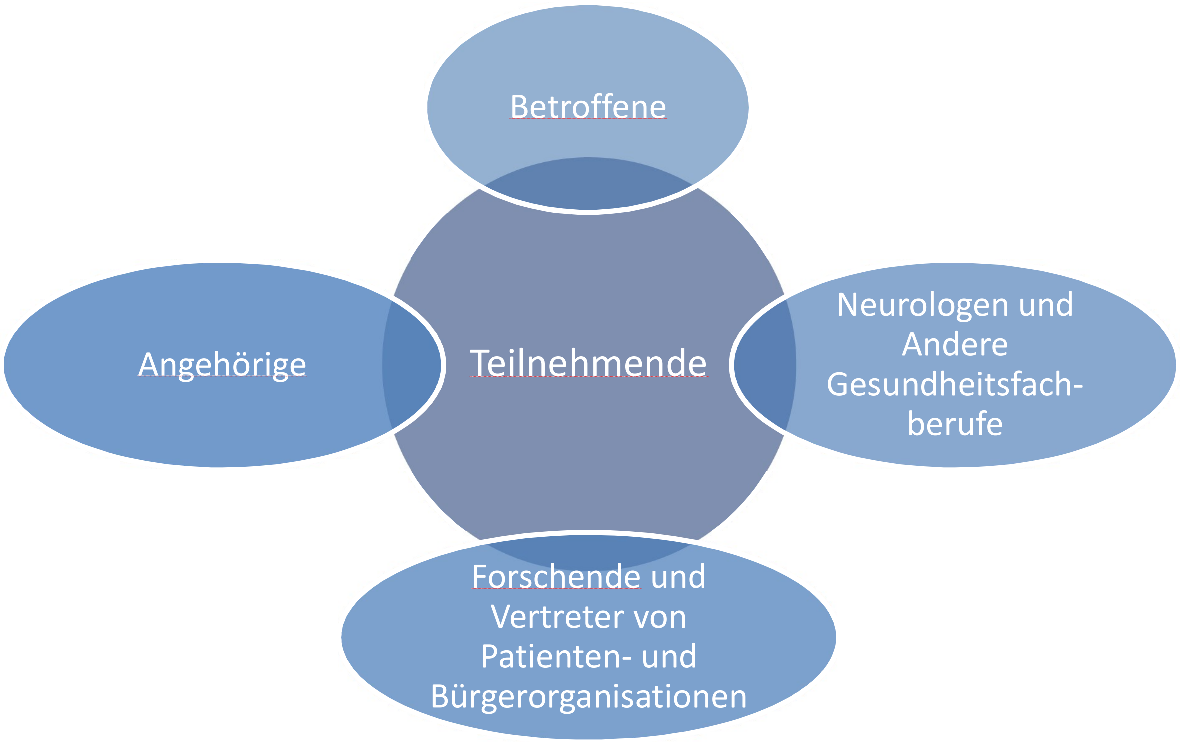

Ziel der Diskussionsrunde

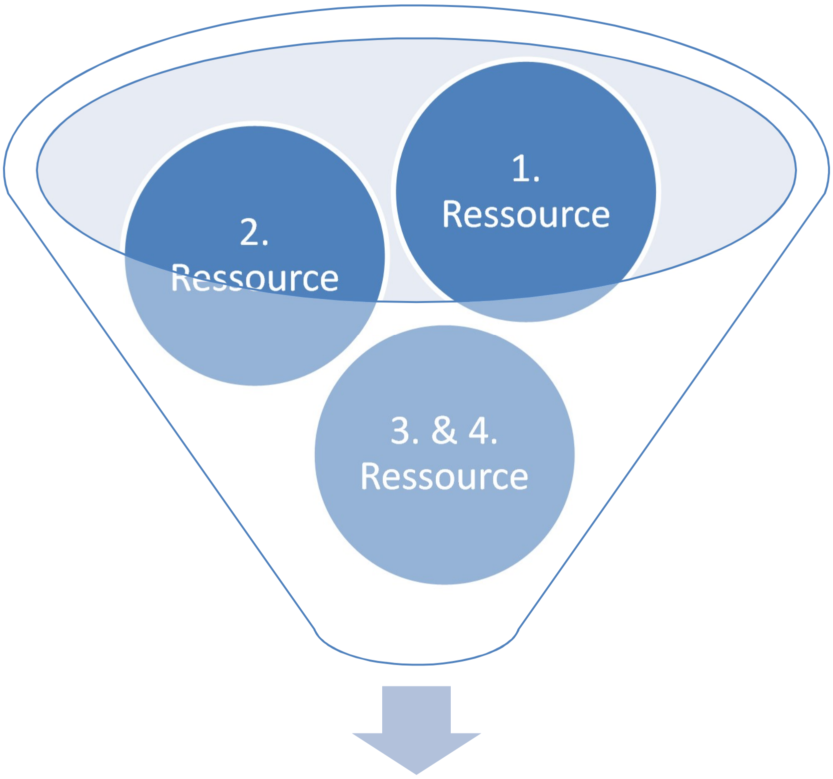

Priorisierung einer Ressource
für Betroffene mit sekundär progredienter Multipler Sklerose (SPMS)

Vier mögliche Ressourcen

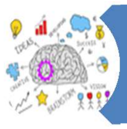

A) Metakognitives und alltagsrelevantes Training

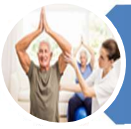

B) Erweitertes Physiotherapieprogramm

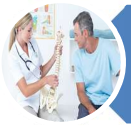

C) Förderung der Patientenbeteiligung an der Versorgung

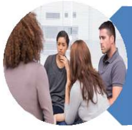

D) Psychologische Unterstützung “READY for MS”

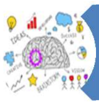

A) Metakognitives und alltagsrelevantes Training

Metakognitives und alltagsrelevantes Training für MS Patienten (MaTiMS)

- Ziel: Behandlung der wichtigsten neuropsychiatrischen Beeinträchtigungen bei MS → Gedächtnis, Aufmerksamkeit, Depression, Fatigue (Müdigkeit), Stress und soziale Einfühlung
- Sitzungen in Kleingruppen à 5-8 Teilnehmenden in 6 Modulen (Dauer: ca. 90 Minuten, 1 mal pro Woche)
- Inhalte

– psycho-edukativer Teil: Vermittlung von Informationen zu typischen Symptomen, epidemiologischen Daten und Faktoren, die zu diesen Symptomen führen sowie zu erforschten Therapiemöglichkeiten

– interaktiven Teil mit Beispielen und Übungen: Erläuterung möglicher Fehlleistungen oder falscher Ansätze beim Umgang mit Defiziten + Vorschläge für bessere Bewältigungsstrategien, Gedanken und Konzepte + direkter Austausch mit den Teilnehmenden

– Angehörige unterstützen das Training durch Einbezug und Feedback in Hausaufgaben

Computerbasiertes Arbeitsgedächtnistraining

– Kognitives Computertraining (Bsp. BrainStim) von Aufmerksamkeit, sprachlichem und räumlichem Gedächtnis

– 2 Mal pro Woche à 45 Minuten für 6 Wochen

Computergestütztes und webbasiertes Begleitprogramm

- Elektronische Plattform für einen personalisierten Pflegeplan, die darin vorgesehenen Besuche und Behandlungen
- Jeder Beteiligter (med. Fachpersonal, Patient und Angehörige) bekommt Zugang zur Plattform
- Über die Plattform erhält der Patient Benachrichtigungen über Datum / Uhrzeit / Ort / Therapien

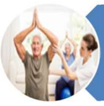

B) Erweitertes Physiotherapieprogramm

Intensive Phase

- Ziel: Verbesserung der Gehfähigkeit und Verringerung der Fatigue (Müdigkeit)
- Zirkeltraining; Dauer: 8 Wochen / 24 Sitzungen (3 Sitzungen pro Woche, 45-60 Minuten)

– Laufbandtraining mit Geh- und Gleichgewichtsübungen

– Gleichgewichtsübungen, die bezüglich der Beschaffenheit der Unterstützungsfläche, der sensorischen Systeme (z.B. geschlossene Augen) und der Bewegungen von Kopf und Augen variiert werden

– Kräftigung geschwächter Muskulatur, insbesondere der Streckmuskeln der unteren Gliedmaßen, der vorderen Schienbeinmuskeln und der Gesäßmuskulatur.

– Durchführung ambulant mit kooperierenden Physiotherapiepraxen (oder im UKE) in Einzelsitzungen

Extensive Phase

- Konzentration auf gleiche Bereiche, wie in der intensiven Phase
- Durchführung der Übungen selbstständig und mit Hilfe eines Therapeuten im Wechsel + telefonische Absprachen
- Dauer: 16 Wochen / 32 Sitzungen (2 Sitzungen pro Woche à 30-45 Minuten) → gliedert sich in 4 Phasen von jeweils 4 Wochen

Motivierende Komponente

- Zur Förderung der aktiven Beteiligung des Patienten werden zusammen mit dem Therapeuten Behandlungsziele definiert, die die Leistungsfähigkeit, Motivation sowie die externen Möglichkeiten des Patienten berücksichtigen
- Die motivierende Komponente beginnt in der Intensivphase und erstreckt sich über die gesamte extensive Phase

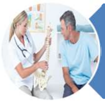

C) Förderung der Patientenbeteiligung an der Versorgung

Gesundheitsdienstleister-Schulung zur Patienteneinbeziehung

- Einführungsschulung für 10-15 Teilnehmende mit Vorträgen zu Werten, Fähigkeiten und Prinzipien der Patientenbeteiligung + Übungen
- Erhaltungsphase der Patientenbeteiligung im Arbeitskontext der Gesundheitsdienstleister (Dauer: 6 Monate)
  - eine monatliche E-Mail mit praktischen Ratschlägen
  - ein monatlicher Telefonkontakt mit einem erfahrenen Patientenbetreuer

Eine Website für Menschen mit SPMS

- Inhalt der Webseite wird patientenorientiert aufgebaut:
  - Festlegen der Hauptthemen (z.B. Wechsel / Beendigung der Immuntherapie, Symptome, Lebensstil) durch Fokusgruppentreffen und persönliche Interviews mit SPMS Betroffenen und ihren Angehörigen
  - Systematische Suche nach den wissenschaftlichen Erkenntnissen zu jedem Thema
- Die Struktur der Website besteht aus:
  - Kapiteln mit einer Einführung zu dem jeweiligen Thema sowie versch. Therapieoptionen und deren Nutzen und Risiken
  - Einem Tagebuch, in dem der Patient über die Art und Schwere seiner Symptome und Probleme berichtet:
  - strukturierter Abschnitt mit Fragebögen und offener Abschnitt für Kommentare und Notizen
  - kann ausgedruckt werden, um sich darüber während der Sprechstunden im MS-Zentrum auszutauschen

frontiers

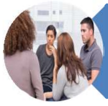

D) Psychologische Unterstützung “READY for MS”

Akzeptanz- und Commitmenttherapie (ACT) bei MS (READY for MS)

- Ziel: Verbesserungen in den Bereichen Belastbarkeit, psychische Flexibilität, körperliche Aktivität, Achtsamkeit, subjektives Wohlbefinden, Gesundheitsverhalten und Lebensqualität
- Gruppenprogramm (5-12 Teilnehmende) besteht aus

a) 7 wöchentlichen Modulen à 2,5 Std. mit Psychoedukation, Erlebnisübungen sowie strukturierten Lern- und Übungsaktivitäten

b) Booster-Session 5 Wochen nach dem letzten Modul → Auffrischen der 7 Module + Bearbeiten von Umsetzungsproblemen

Persönliche Psychotherapie (falls erforderlich), Kooperation mit Psychotherapeutenkammer, Entwicklung einer MS-Kontaktliste oder Computergestützte/telefonische Therapie (MS-DEPREXIS)

Computergestütztes und webbasiertes Begleitprogramm

- Elektronische Plattform für einen personalisierten Pflegeplan, die darin vorgesehenen Besuche und Behandlungen
- Jeder Beteiligter (med. Fachpersonal, Patient und Angehörige) bekommt Zugang zur Plattform
- Über die Plattform erhält der Patient Benachrichtigungen über Datum / Uhrzeit / Ort / Therapien

Bewertung der Ressourcen nach den Kriterien:
Relevanz, Angemessenheit und einfache Implementierung

1. **Relevanz:** Wie relevant (bedeutsam/wichtig) ist die Ressource für Betroffene mit SPMS?
2. **Angemessenheit:** Ist die Ressource, so wie sie geplant ist angemessen, um den Bedarf von Betroffenen mit SPMS zu erfüllen?
3. **Einfache Implementierung:** Ist die Ressource einfach im Gesundheitswesen und in der Versorgung umsetzten?

frontiers

Ablauf

**15:00 – 15:45**	Einführung
**15:45 – 16:00**	Einzelranking
**16:00 – 16:15**	Kaffeepause
**16:15 – 17:00**	Einzelgruppendiskussionen
**17:00 – 17:15**	Kaffeepause
**17:15 – 18:00**	Plenarsitzung

Rankingsystem

**A) Metakognitives Training**			**B) Erweitertes Physiotherapieprogramm**			**C) Patienten-beteiligung**			**D) Psychologische Unterstützung**		
R	A	E	R	A	E	R	A	E	R	A	E
**R = Relevanz**<br>**A = Angemessenheit**<br>**E = Einfache Implementierung**											

Bitte tragen Sie Zahlen von 1 bis 4 ein, wobei die Zahlen folgendes bedeuten:

4
- am relevantesten
- am angemessensten
- am einfachsten zu implementieren

3
- eher relevant
- eher angemessen
- eher einfach zu implementieren

2
- weniger relevant
- weniger angemessen
- weniger implementierbar

1
- nicht relevant
- nicht angemessen
- nicht implementierbar

Ergebnisse der Einzelgruppendiskussionen

Reihenfolge der Priorisierung				
	A) Metakognitives Training	B) Erweitertes Physiotherapieprogramm	C) Patientenbeteiligung	D) Psychologische Unterstützung
**Betroffene**	Platz 1	Platz 2	Platz 3	Platz 1
**Angehörige**	Platz 3	Platz 2	Platz 4	Platz 1
**Forschende und Vertreter von Patienten- und Bürgerorganisationen**	Platz 3	Platz 2	Platz 3	Platz 1
**Neurologen und Andere Gesundheitsfachberufe**	Platz 2	Platz 1	Platz 3	Platz 1

Ergebnis der Plenarsitzung

Nach einer Diskussion der Prioritätenlisten der Einzelgruppen stimmten alle Teilnehmer der folgenden Prioritätenliste zu:

1. D) Psychologische Unterstützung "READY for MS"
2. B) Erweitertes Physiotherapieprogramm
3. A) Metakognitives und alltagsrelevantes Training
4. C) Förderung der Patientenbeteiligung an der Versorgung

Folgende Punkte wurden während der Diskussion erwähnt:

- Psychologische Unterstützung ist am wichtigsten, denn sie ist Grundlage für Achtsamkeit, akzeptanzbasierte Strategien, Energie und Engagement, um fähig zu sein, die anderen genannten Maßnahmen (z.B. Physiotherapie) zu nutzen.
- Tätige im Gesundheitsbereich sollten die Rolle eines Coaches (was zu mehr Autonomie führt) und nicht die eines Leiters übernehmen.
- Patienten wünschen sich die Kombination von Psychologie-, Kognitions- und Physiotherapieelementen
- Da das Aufsuchen eines Psychologen immer noch für einige Menschen mit Stigmatisierung verbunden ist und gemieden wird, wäre es sinnvoll, die psychologischen Unterstützung innerhalb einer Lifestyle-Intervention anzubieten. Hierbei hätten die Betroffenen die Möglichkeit, Schwerpunkte (z.B. auf Psychotherapie) zu setzen, würden aber nicht nur zur reinen Psychotherapie gehen.
- Physiotherapie ist sehr wichtig, da sie auch einen Einfluss auf Kognition und Fatigue hat. Die Maßnahme, so wie sie geplant ist (Training 3-mal pro Woche), ist aber schwierig umzusetzen.
