# Supplementary material for: Construction of a User-Led Resource for People Transitioning to Secondary Progressive Multiple Sclerosis: Results of an International Nominal Group Study
Source: Front Neurol. 2020 Aug 18;11:798. doi: 10.3389/fneur.2020.00798 (PMC7461961; doi:10.3389/fneur.2020.00798)
Supplement: Supplementary file 3 [file Data_Sheet_3.PDF]

Supplementary File 3. Consolidated criteria for reporting qualitative studies (COREQ)

Consolidated criteria for reporting qualitative studies (COREQ): 32-item checklist

No. Item	Guide questions/description	Reported on Page #
**Domain 1: Research team and reflexivity**		
Personal Characteristics		
1. Interviewer/facilitator	Which author/s conducted the inter view or focus group?	Italy: A Solari, AM Giovannetti, A Giordano, R Quintas, S Alfieri.<br><br>Germany: A Barabasch, C Muche-Borowski, K Riemann-Lorenz, S Lau.
2. Credentials	What were the researcher's credentials? E.g. PhD, MD	Italy<br>• A Solari: M.Sc.<br>• AM Giovannetti: M.Sc.<br>• A Giordano: M.Sc.<br>• R Quintas: M.Sc.<br>• S Alfieri: Ph.D<br><br>Germany<br>• A Barabasch: M.Sc.<br>• C Muche-Borowski: Dr. rer. Biol. Hum.<br>• K Riemann-Lorenz: M.Sc.<br>• S Lau: educ. diploma
3. Occupation	What was their occupation at the time of the study?	• A Solari is the head of the Neuroepidemiology Unit, Fondazione IRCCS Istituto Neurologico Carlo Besta, Milan, Italy<br>• AM Giovannetti is a psychologist/researcher at the Fondazione IRCCS Istituto Neurologico Carlo Besta, Milan, Italy, and Ph.D student at the University of Queensland, Australia (FISM scholarship)<br>• A Giordano is a psychologist/researcher at the Fondazione IRCCS Istituto Neurologico Carlo Besta, Milan, Italy, and Ph.D student at the University of Turin, Turin, Italy<br>• R Quintas is a psychologist/researcher

		at the Fondazione IRCCS Istituto Neurologico Carlo Besta, Milan, Italy<br>• S Alfieri is a researcher at Unit of Clinical Psychology, Foundation IRCCS Istituto Nazionale per la Cura dei Tumori, Milan, Italy<br>• A Barabasch is an associate research and Ph.D student at the Institute of Neuroimmunology and Multiple Sclerosis, UKE Hamburg, Germany<br>• C Muche-Borowski is research associate at the Institute of General Medicine, UKE Hamburg.Germany<br>• K Riemann-Lorenz is a research associate and PhD student at the Institute of Neuroimmunology and Multiple Sclerosis, UKE Hamburg, Germany<br>• S Lau is part of the clinical care team of the MS outpatient clinic, UKE Hamburg, Germany
4. Gender	Was the researcher male or female?	Two males, seven females
5. Experience and training	What experience or training did the researcher have?	Moderators had long lasting experience (>10 years)<br>All facilitators had over 2 years experience except one person who was specially trained for this study.
Relationship with participants		
6. Relationship established	Was a relationship established prior to study commencement?	Materials and Methods: Procedure
7. Participant knowledge of the interviewer	What did the participants know about the researcher? e.g. personal goals, reasons for doing the research	Materials and Methods: Procedure
8. Interviewer characteristics	What characteristics were reported about the interviewer/facilitator? e.g. Bias, assumptions, reasons and interests in the research topic	N.R.
**Domain 2: study design**		
Theoretical framework		
9. Methodological orientation and Theory	What methodological orientation was stated to underpin the study? e.g. grounded theory, discourse analysis, ethnography, phenomenology, content analysis	Thematic analysis (Materials and Methods: Analysis)
Participant selection		

10. Sampling	How were participants selected? e.g. purposive, convenience, consecutive, snowball	Purposive (Materials and Methods: Participants)
11. Method of approach	How were participants approached? e.g. face-to-face, telephone, mail, email	Materials and Methods: Procedure
12. Sample size	How many participants were in the study?	
13. Non-participation	How many people refused to participate or dropped out? Reasons?	Results (first paragraph)
Setting		
14. Setting of data collection	Where was the data collected? e.g. home, clinic, workplace	Clinic (Materials and Methods: Procedure)
15. Presence of non-participants	Was anyone else present besides the participants and researchers?	No
16. Description of sample	What are the important characteristics of the sample? e.g. demographic data, date	Table 1
Data collection		
17. Interview guide	Were questions, prompts, guides provided by the authors? Was it pilot tested?	Not applicable
18. Repeat interviews	Were repeat inter views carried out? If yes, how many?	Not applicable
19. Audio/visual recording	Did the research use audio or visual recording to collect the data?	Audio-recording (Materials and Methods: Procedure)
20. Field notes	Were field notes made during and/or after the interview or focus group?	Yes
21. Duration	What was the duration of the inter views or focus group?	Results (first paragraph)
22. Data saturation	Was data saturation discussed?	Not applicable
23. Transcripts returned	Were transcripts returned to participants for comment and/or correction?	Yes (Materials and Methods: Analysis)
**Domain 3: analysis and findings**		
Data analysis		
24. Number of data coders	How many data coders coded the data?	Materials and Methods: Analysis
25. Description of the coding tree	Did authors provide a description of the coding tree?	No

26. Derivation of themes	Were themes identified in advance or derived from the data?	Both
27. Software	What software, if applicable, was used to manage the data?	Not applicable
28. Participant checking	Did participants provide feedback on the findings?	Materials and Methods: Analysis
Reporting		
29. Quotations presented	Were participant quotations presented to illustrate the themes/findings? Was each quotation identified? e.g. participant number	Quotations were presented and identified (Results: Consensus routing)
30. Data and findings consistent	Was there consistency between the data presented and the findings?	Yes (Results: Consensus routing)
31. Clarity of major themes	Were major themes clearly presented in the findings?	Yes (Results: Consensus routing)
32. Clarity of minor themes	Is there a description of diverse cases or discussion of minor themes?	Diverse cases/groups (Results: Consensus routing)
